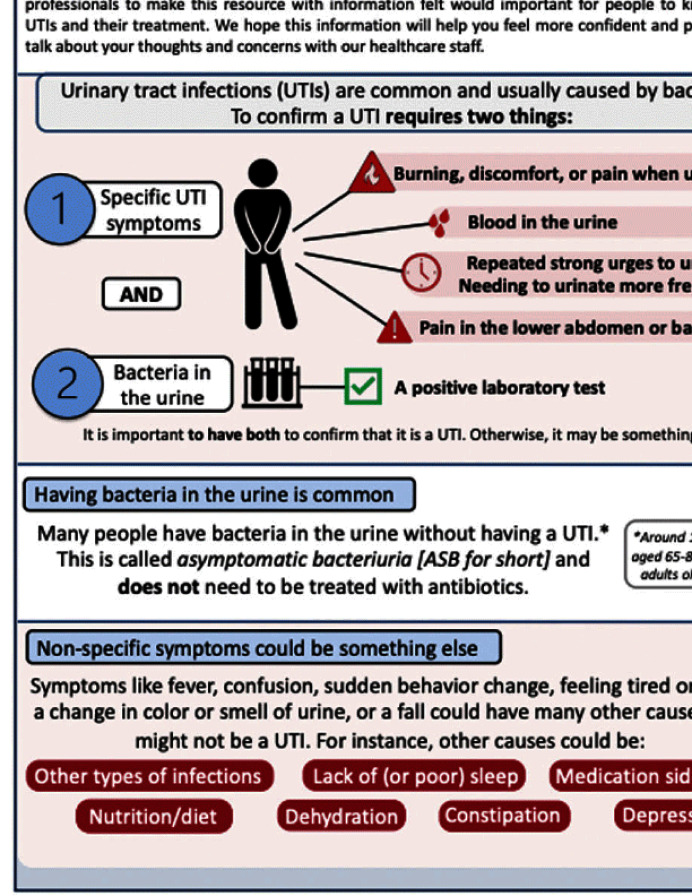# User-Centered Education for Patients/Caregivers about Urinary Tract Infections, Asymptomatic Bacteriuria, and Antibiotics

**DOI:** 10.1017/ash.2024.101

**Published:** 2024-09-16

**Authors:** Alistair Thorpe, Karen Fong, Hannah Hardin, Brandi Muller, Julie Szymczak, Andrea White, Valerie Vaughn

**Affiliations:** University of Utah; University of Utah Health; University of Utah School of Medicine; Division of General Internal Medicine

## Abstract

**Background:** Older adults (aged ≥65) are at high risk of harm from overdiagnosis and overtreatment of urinary tract infections (UTIs) with antibiotics. Involving patients/caregivers in their antibiotic treatment decisions has potential to improve prescribing. To engage effectively, patients/caregivers must have sufficient knowledge about UTIs, asymptomatic bacteriuria (ASB: bacteria in the urine without signs of UTI), and antibiotics and opportunities to share their concerns and treatment preferences with healthcare staff. Patient education is one of the core elements of antibiotic stewardship recommended by the Centers for Disease Control and Prevention but, there are few resources for patients/caregivers about UTIs and antibiotics, leaving a knowledge gap as to what effective patient/caregiver antibiotic education for UTIs looks like. We sought to better understand the perspectives of patients/caregivers at high-risk of antibiotic overuse for UTIs and create an educational leaflet on UTIs, antibiotics, and ASB. **Method:** Between 11/2022 and 03/2023, we conducted virtual semi-structured interviews with patients ≥65yrs who had experienced UTI and caregivers about their needs, experiences, and preferences for educational support. Interviews lasted ~1 hour. Audio recordings were transcribed verbatim. NVivo software managed the data, which we analyzed using thematic analysis. **Results:** We conducted 9 interviews (5 patients, 4 caregivers). Interviewees expressed desire to be involved in their treatment decisions and learn more about antibiotics and alternative strategies (themes shown in Figure 1). Reported reasons for limited involvement in decisions included lacking the knowledge and confidence to ask questions, emotional factors (e.g., embarrassment/stress), deference to healthcare staff, and time constraints. Healthcare staff behaviors were described both as barriers (e.g., assertive treatment decisions) and facilitators (e.g., effective communication) of patient/caregiver engagement. Interviewees were eager for printed and digital educational support that could provide tailored content to help improve their knowledge and prepare for future conversations with healthcare staff. From this feedback we developed an educational leaflet (Figure 2). **Conclusions:** Involving patients/caregivers in antibiotic treatment decisions represents an opportunity to intervene before patients experience antibiotic overuse. Our findings offer important insights on patient/caregiver’ educational needs and preferences as well as perceived barriers to engaging in antibiotic treatment decisions for UTI. We used these insights to inform the development of educational materials about UTIs, ASB, and antibiotics for patients/caregivers and plan to test their use through multiple mediums with tailoring for unique patient needs, experiences, and backgrounds.

**Figure:**
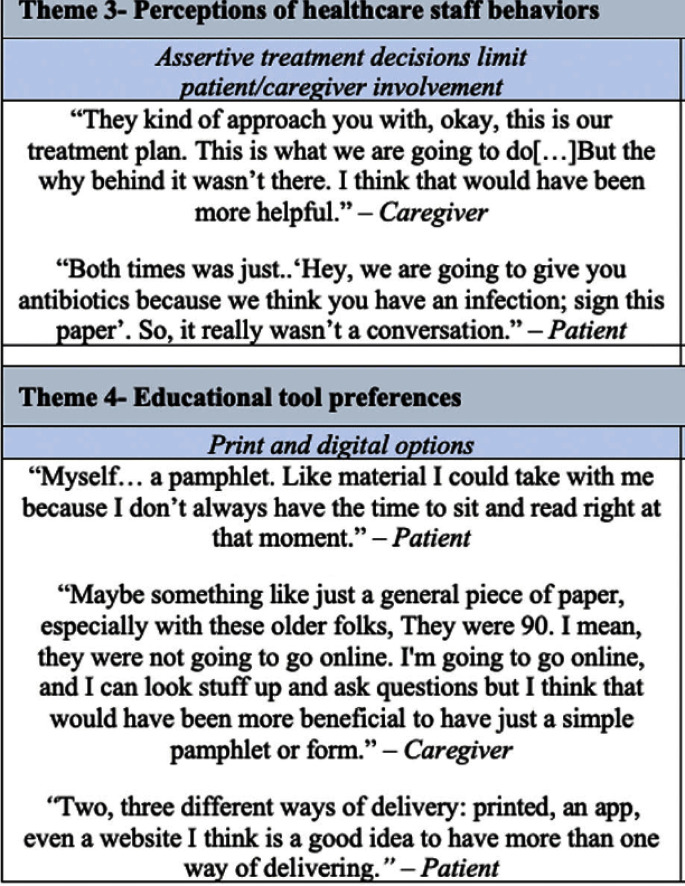

**Figure:**